# *Prunus persica* plant endogenous peptides PpPep1 and PpPep2 cause PTI-like transcriptome reprogramming in peach and enhance resistance to *Xanthomonas arboricola* pv. *pruni*

**DOI:** 10.1186/s12864-021-07571-9

**Published:** 2021-05-18

**Authors:** Laura Foix, Anna Nadal, Maja Zagorščak, Živa Ramšak, Anna Esteve-Codina, Kristina Gruden, Maria Pla

**Affiliations:** 1 Institute for Agricultural and Food Technology, Universitat de Girona, Campus Montilivi (EPS-1), 17003 Girona, Spain; 2 Department of Biotechnology and Systems Biology, National Institute of Biology, Večna pot 111, 1000 Ljubljana, Slovenia; 3 CNAG-CRG, Centre for Genomic Regulation, Barcelona Institute of Science and Technology, 08028 Barcelona, Spain; 4 Universitat Pompeu Fabra (UPF), Barcelona, Spain

**Keywords:** Plant elicitor peptide (Pep), Plant defense, *Prunus*, RNA sequencing, Differential network analysis, Gene set enrichment analysis

## Abstract

**Background:**

Rosaceae species are economically highly relevant crops. Their cultivation systems are constrained by phytopathogens causing severe losses. Plants respond to invading pathogens through signaling mechanisms, a component of which are of them being plant elicitor peptides (Peps). Exogenous application of Peps activates defense mechanisms and reduces the symptoms of pathogen infection in various pathosystems. We have previously identified the Rosaceae Peps and showed, in an ex vivo system, that their topical application efficiently enhanced resistance to the bacterial pathogen *Xanthomonas arboricola* pv. *pruni* (Xap).

**Results:**

Here we demonstrate the effectiveness of *Prunus persica* peptides PpPep1 and PpPep2 in protecting peach plants in vivo at nanomolar doses, with 40% reduction of the symptoms following Xap massive infection. We used deep sequencing to characterize the transcriptomic response of peach plants to preventive treatment with PpPep1 and PpPep2. The two peptides induced highly similar massive transcriptomic reprogramming in the plant. One hour, 1 day and 2 days after peptide application there were changes in expression in up to 8% of peach genes. We visualized the transcriptomics dynamics in a background knowledge network and detected the minor variations between plant responses to PpPep1 and PpPep2, which might explain their slightly different protective effects. By designing a *P. persica* Pep background knowledge network, comparison of our data and previously published immune response datasets was possible.

**Conclusions:**

Topical application of *P. persica* Peps mimics the PTI natural response and protects plants against massive Xap infection. This makes them good candidates for deployment of natural, targeted and environmental-friendly strategies to enhance resistance in *Prunus* species and prevent important biotic diseases.

**Supplementary Information:**

The online version contains supplementary material available at 10.1186/s12864-021-07571-9.

## Background

Endogenous peptide elicitors (Peps) are a type of DAMP (damage-associated molecular pattern) first identified in *A. thaliana* by Huffaker and colleagues [1]. These 20–23 amino acid long peptides mature from the C-terminal portion of their larger precursor proteins called PROPEPs and are recognized by leucine-rich repeat (LRR) receptor-like kinases known as Pep receptors (PEPRs) [2–4]. As well as bacterial flagellin or EF-Tu perception, Peps not only trigger but also amplify the innate immunity of plants against pathogens [5].

To date, a number of plant peptides have been identified as defense-related signals. Up to eight PROPEP and Pep genes have been described in *A. thaliana* and other Brassicaceae [1, 2], seven have been found in *Zea mays*, three in *Oryza sativa* [6, 7], and between one and three in many Rosaceae, Fabaceae and Solanaceae species [3, 7–9]. We have previously identified Pep sequences from 36 economically relevant Rosaceae species, with two tribe-specific Pep types per plant, Pep1 and Pep2 (Amygdaleae) and Pep3 and Pep4 (Pyreae) [10]. Among studied species, only one or two PEPRs have been observed [3, 7, 11–13]. In the same Rosaceae species we identified two PEPRs, PEPR1a and PEPR1b, with higher homology to AtPEPR1 than AtPEPR2 [10]. Pre-treatment of *A. thaliana*, *Zea mays* and *Prunus* spp. plants with Peps significantly improves their resistance to pathogens, including bacteria and fungi, as well as to herbivores [1, 3, 6, 7, 9, 12]. This resistance is triggered by activating defense responses e.g. ethylene response factors (ERFs) and pathogenesis-related (PR) genes, which were up-regulated after topical application of *Prunus persica* Peps (PpPeps) onto *Prunus* spp. leaves [9].

Similarly to flg22 (N-terminal part of flagellin) recognition by FLS2 (flagellin sensing 2), upon Pep perception, PEPRs interact with the coreceptor BAK1 (Brassinosteroid Receptor-Associated Kinase-1) [12, 14, 15] to induce a typical innate immunity–like response [16]. Studies in *A. thaliana* indicates that Pep-PEPR complexes are internalized via clathrin-mediated endocytosis (CME) [17]. In a matter of seconds this leads to the activation of downstream defense cascades including ion fluxes across the plasma membrane, such as an increase in Ca^2+^ influx [18], followed by phosphorylation of mitogen-activated protein kinases via MAP kinase cascades [19, 20]. Activation of 1-amin-cyclopropane-1-carboxylate (ACS) synthase is responsible for stomatal closure 30 min after elicitor perception [6, 21]. Biosynthesis of defense-related molecules such as ethylene (ET), salicylic acid (SA) and jasmonic acid (JA) is enhanced, as well as new PEPRs proteins replacing internalized ones [17]. An extensive transcriptional reprogramming is activated in a matter of hours [22], and cell wall re-modelling and synthesis of antimicrobial products is triggered within days [23, 24]. Interestingly, Peps seem to have positive feedback since AtPep1 induces expression of its own precursor, so amplifying the pattern-triggered immunity (PTI) response [1, 25]. This response has been specially studied in AtPeps, as they not only play a critical role in immunity, but may also be involved in development, and other biological processes from germination to flowering and seed production [25–27]. However, their specific multiple functions and tissue-linked activities are still to be determined.

Stone-fruits (peaches, nectarines, cherries and plums) are among the most important fruit crops in temperate areas with a global annual production of 46 million tonnes [28]. However, several abiotic and biotic diseases, such as bacterial spot and canker of stone-fruits caused by *Xanthomonas arboricola* pv. *pruni* (hereafter, Xap), limit the production. New insights into environmentally-friendly disease control are needed in order to replace copper derivative bactericides and antibiotics. Our previous ex vivo assays proved that protection of *P. persica* leaves against Xap infection using PpPeps [9, 10]. With the final aim of defining the potential of commercial application of Peps as tool for enhancement of the resistance of Rosaceae plants to pathogens, here we determined the efficacy of PpPeps in vivo. In addition, we used transcriptome deep sequencing to further analyze the peach response to topical application of peach Peps, specifically at doses that efficiently protect *P. persica* leaves against Xap infection.

## Results

### PpPep1 and PpPep2 protect *Prunus* plants against the bacterial pathogen Xap

We previously used an ex vivo approach to demonstrate that topical application of PpPep1 and PpPep2 protected *P. persica* x *P. dulcis* leaves against the phytopathogen Xap. Optimal efficiencies were on treatment with 0.1 and 1 μM doses 24 and 48 h prior to pathogen challenge. Here we assessed the efficiency of treatment with the same peptides to protect *Prunus* against the same phytopathogen in plants grown in the greenhouse. Intact *P. persica* x *P. dulcis* plants were treated with water or the chemically synthesized PpPep1 and PpPep2, 24 h before the inoculation with 10^8^ cfu/mL Xap.

**Fig. 1:**
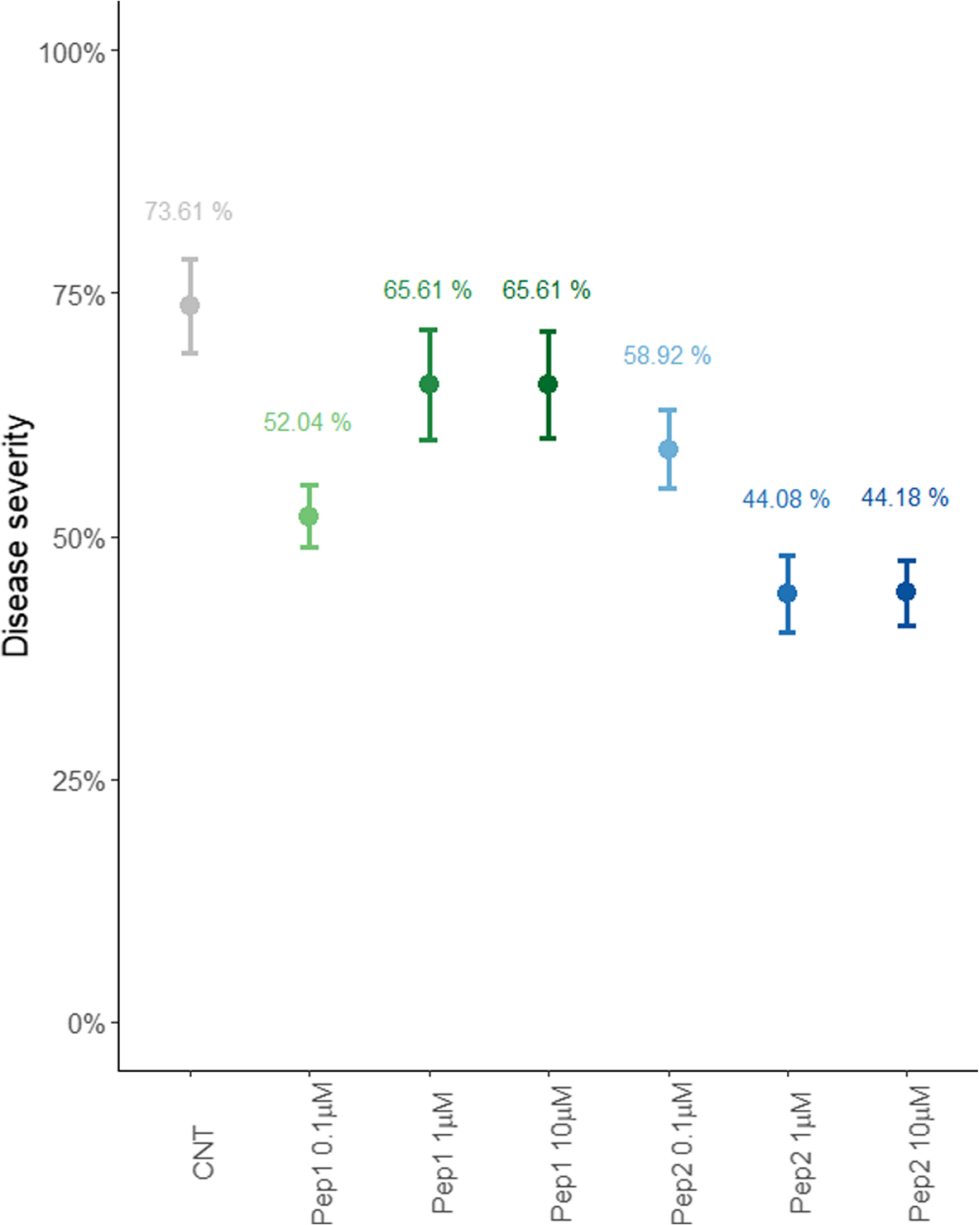
Disease severity in *P. persica* x *P. dulcis* plants treated with different concentrations of PpPep1 and PpPep2 for 24 h, prior to infection with 10^8^ cfu/mL Xap, in relation to untreated plants (CNT). Data taken nine days after infection. Mean values and error bars shown. Sample size: three biological replicates, for each measurement on the tree leaves; for each of the six treatments (Pep1: green, Pep2: blue) and one control (grey)

Leaves treated with water showed the typical symptoms of bacterial spot infection. About 1 week after Xap inoculation, infection became apparent as small, pale-green to yellow, circular or irregular areas with a light tan center. Over time, symptoms developed, with an increase in number and size of the spots, becoming more angular in shape and brown or black with a yellow halo. The lesions merged to cover up to a 50% of the leaf surface 4 weeks post bacterial inoculation, when leaves fell off from the plant. In contrast, application of PpPep1 and PpPep2 resulted in a lower percentage of affected leaf surface at all time points assessed i.e. up to 3 weeks after infection. Figure 1 shows these differences 9 days after inoculation, as it was confirmed using Kruskal-Wallis post-hoc pairwise comparisons (IMB SPSS Statistics 25, *p* < 0.05). This indicates that these treatments efficiently protected *Prunus* plants from Xap. Moreover, there was a dose-dependent effect on plant protection. For PpPep2, 1 and 10 μM doses resulted in 40% reduction of the Xap symptoms 9 days after infection (Fig. 1). The maximum protection with PpPep1 was achieved at 0.1 μM doses. Thus, 0.1 μM PpPep1 and 1 μM PpPep2 concentrations were selected for further experiments.

### RNA-Seq characterization of peach transcriptomic response to PpPep1 and PpPep2

To characterize the peach response to preventive treatment with PpPep1 and PpPep2 we treated juvenile *P. persica* plants with the most effective dose of each peptide and sequenced their transcriptomes after 1, 24 and 48 h using untreated plants as control. Data supporting this analysis is available in the Gene Expression Omnibus (GEO) repository, record GSE161802 (https://www.ncbi.nlm.nih.gov/geo/query/acc.cgi?acc=GSE161802).

Additional file 1 shows summary mapping statistics of RNA sequencing to the *P. persica* reference genome. There were on average 55 million 75 bp paired stranded reads, for each experimental replicate. The percentage of 91% paired mapped of reads indicated appropriate quality of the libraries. On average, about 95% of the read pairs aligned to exonic locations in the reference genome, while slightly more than 1% aligned to intronic regions, suggesting new isoforms, presence of pre-mRNA or imprecise transcript length prediction. Less than 5% read pairs aligned to intergenic regions, which might correspond to yet unannotated genes in the peach genome. Other good library quality indicators were that about 25% of genes consumed 25% of reads, the percentage of PCR duplicates (28–36%) and the high proportion of reads (uniquely paired-end mapped reads) mapping to protein coding regions (> 99%) [29]. Of 24,898 genes in the *P. persica* NCBIv2.38 genome, more than 20,500 were covered by at least one read pair in each sample.

Specific RT-qPCR assays were developed to target 19 genes belonging to 18 out of 49 differential functional categories (BINs, Additional file 2) and displaying above 1000 counts at least in one sample. RNA levels were assessed in peach leaf samples obtained as for the RNA-Seq experiments, 0, 1, 24 and 48 h after PpPep1 and PpPep2 topical application. High correlation of differential gene expression between RNA-Seq and RT-qPCR data was observed (Pearson correlation of 0.92), validating the relative quantification performance of RNA-Seq results (Additional file 2).

### PpPep1 and PpPep2 elicit similar transcriptomic responses

**Fig. 2:**
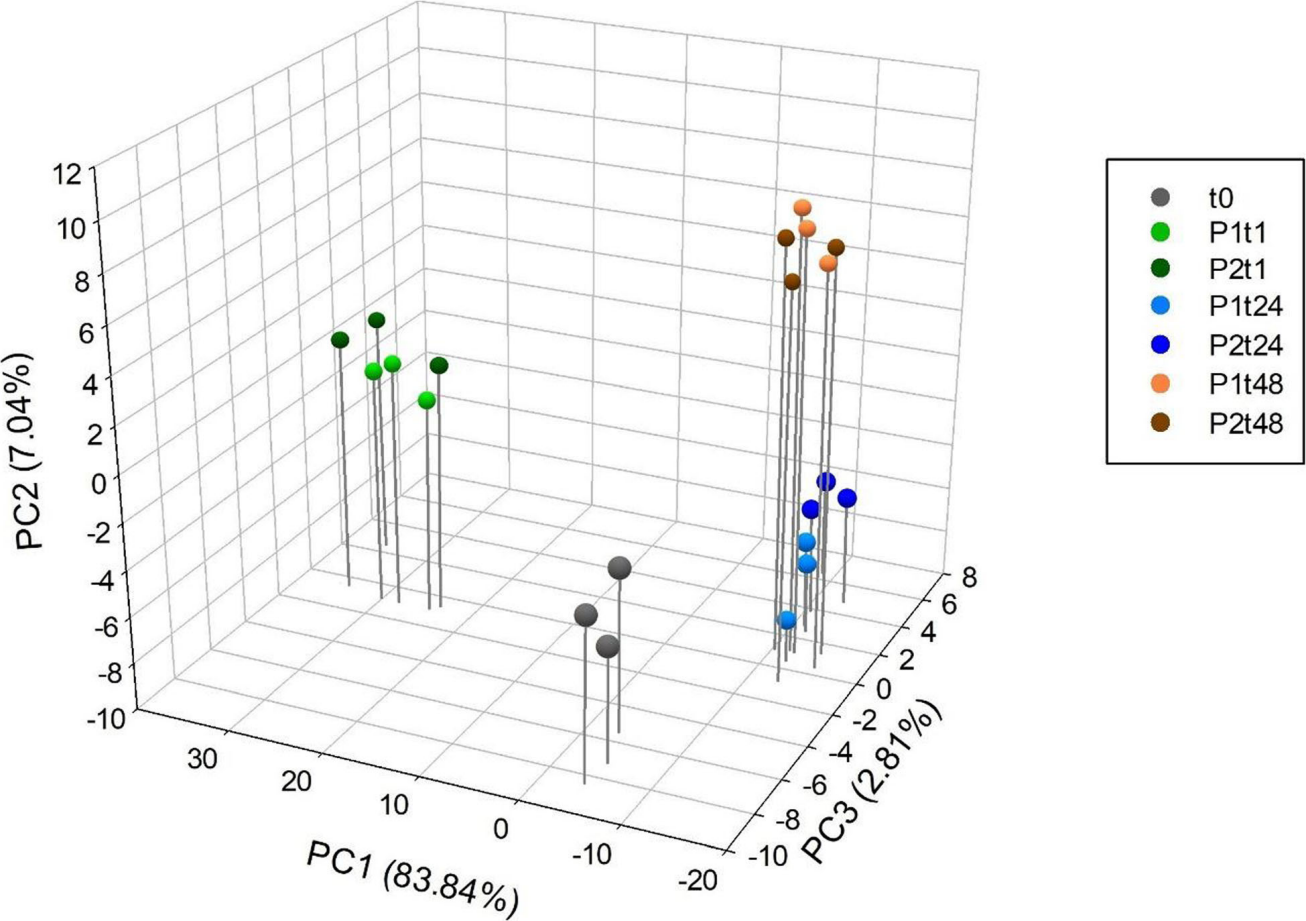
Principal component analysis (PCA) of log2 normalized expression data from the 500 genes showing the highest variance (DESeq2 1.20.0 package, [60]). Three principal components, PC1, PC2 and PC3, with Eigenvalues above 1 explained 83.84, 7.04 and 2.81% of the overall variability, respectively. The peptide treatments are shown in different colors: grey, time zero (t0); light green, 1 h PpPep1 (P1t1); dark green, 1 h PpPep2 (P2t1); light blue, 24 h PpPep1 (P1t24); dark blue, 24 h PpPep2 (P2t24); orange, 48 h PpPep1 (P1t48); brown, 48 h PpPep2 (P2t48). Three biological replicates per sample are shown. Interactive PCA is available at Additional file 3

Principal Component Analysis (PCA) of the processed data (Fig. 2 and Additional file 3) shows that the main transcriptomic changes can be attributed to the treatment time-course condition and not to the specific peptide. The transcriptomes of peach leaves sampled at the same time-points after treatment with either PpPep1 or PpPep2 were not separated across a three-dimensional PCA scatterplot (Fig. 2 and Additional file 3), while PC1, PC2 or PC3 together explained up to 93.69% of the overall data variability.

Differential expression analysis was carried out on the basis of normalized gene reads using DESeq2 and adjusted *p* value (adj. p) threshold < 0.01. To highlight the time-course responses, transcriptomes of plants treated with PpPep1 or PpPep2 for a certain period of time were compared to those corresponding to the previous time-point. In a complementary approach using RT-qPCR, no significant changes in gene expression between mock plants sampled at 0, 1, 24 and 48 h time-points were detected (*p* > 0.01, IBM SPSS statistics 25; Additional file 4).

**Fig. 3:**
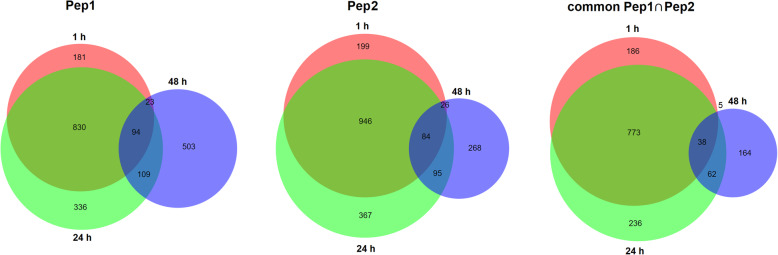
Venn diagrams representing overlapping differentially expressed genes in response to PpPep1 (left panel) and PpPep2 (middle panel), as well as genes commonly regulated in both treatments (right panel). 1 h: 1 h vs. control; 24 h: 24 h vs. 1 h; 48 h: 48 h vs. 24 h; ∩: intersection i.e. common DEGs between comparisons. Additional file 6 shows Venn diagrams considering upregulated and downregulated gene numbers

Figure 3 and Additional file 5 summarize differentially expressed genes (DEG) results in response to PpPeps. We identified a total of 2076 genes exhibiting differential expression upon PpPep1 application and 1985 for PpPep2, accounting for 8% of the *P. persica* genes. Overall, 6% of peach genes (1464) were regulated in response to both peptides. The similitude of response to PpPep1 and PpPep2 reached 80 and 89% of the total amount of DEG and tended to decrease with time, to 74 and 68% DEG 1 day after treatment, and to 69 and 57% DEG 2 days after treatment.

One hour after Pep application there was regulation of ca. 5% peach genes, with more than 90% upregulated (Fig. 3 and Additional files 5 and 6). One day after Pep treatment DEG also encompassed ca. 5% of the total *P. persica* genes, among which ca. 80% were downregulated (Fig. 3, Additional files 5 and 6). For about 20% DEG, which accounts for 1.2% of the peach genes, regulation was first detected 1 day after Pep application (Fig. 3). Another 24 and 14% DEG, for PpPep1 and PpPep2 respectively, were first detected 2 days after treatment (Fig. 3). This set of late DEG agrees with PC2 (Fig. 2 and Additional file 3).

**Fig. 4:**
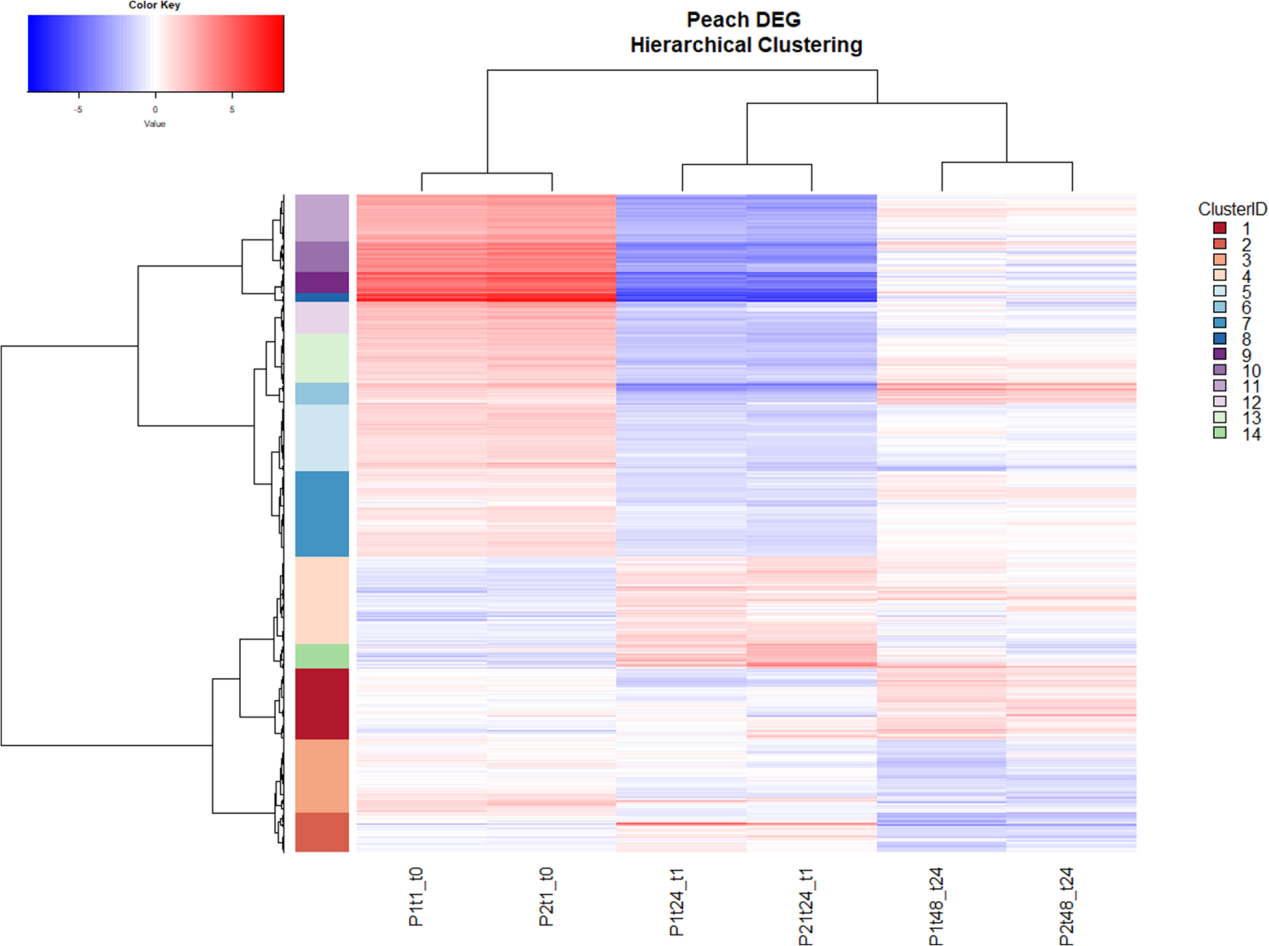
Hierarchical clustering analysis using Ward’s method and Euclidean distance of genes with differential expression (adj. *p* < 0.01, |log2FC| > 1.0) in at least one comparison: P1t1 vs. t0, P2t1 vs. t0, P1t24 vs. t1, P2t24 vs. t1, P1t48 vs. t24, and P2t48 vs. t24. P1: treatment with PpPep1; P2: treatment with PpPep2; time points: t0, t1, t24, and t48 for non-treated, and 1 h, 24 h and 48 h treatments, respectively. The dash corresponds to vs.. The color scale, red to blue, represents highly positive to highly negative log2FC, white corresponding to 0. Clustering into 14 partitions is shown on the left

The expression patterns of all 2542 genes with differential expression in at least one treatment (i.e. PpPep1 or PpPep2 application for 1, 24 or 48 h) are represented as a cluster heat map (Fig. 4 and Additional file 7).

### Analysis of the peach transcriptomic response to PpPeps on the level of processes

RNA-Seq gene expression data was subjected to gene set enrichment analysis to assist biological interpretation of transcriptome changes in response to Peps running the Singular Enrichment Analysis (SEA) within the GSEA toolkit [30]. As shown in Table 1 and Additional files 8 and 9, 49 BINs were enriched at least in one condition, comprising of 1194 enrichment contributor genes. Numerous BINs and SUBBINs showed upregulation 1 h after peptide application and downregulation 1 day later, in response to both PpPep1 and PpPep2, describing an initial and transient transcriptomic response to PpPeps. Another set of BINs were enriched 2 days after treatment with Peps.

**Table 1:**
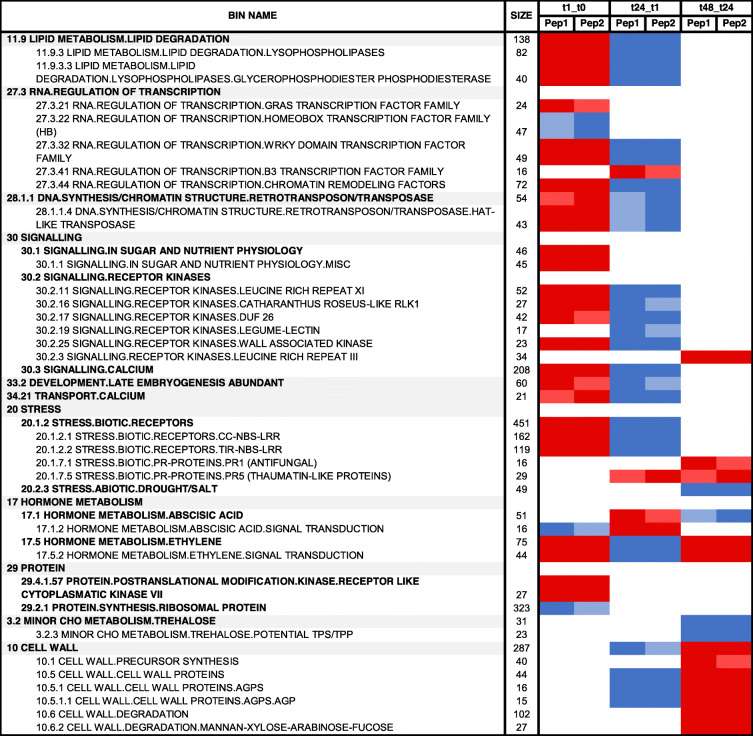
Time-course transcriptome response of *P. persica* leaves to treatment with PpPep1 and PpPep2

Gene set enrichment analysis was carried out using GSEA and adjusted *p* < 0.01. For every peptide (PpPep1 and PpPep2), samples treated for 1, 24 and 48 h were compared to those corresponding to the previous time-point [first vs. second time (t) condition in the first row]. Red and blue indicate upregulation and downregulation, respectively, of the genes contributing to each bin. Bright red and blue indicate statistically significant enrichment. Blank, light red and light blue cells indicate the absence of statistically significant enrichment. Size indicates the number of genes in the gene set after filtering out those not in the expression dataset. Additional file 8 shows the data for all BINs and comparisons and Additional file 9 has detailed information about all genes contributing to each BIN enrichment

### Dynamic visualization of RNA-Seq results

**Fig. 5:**
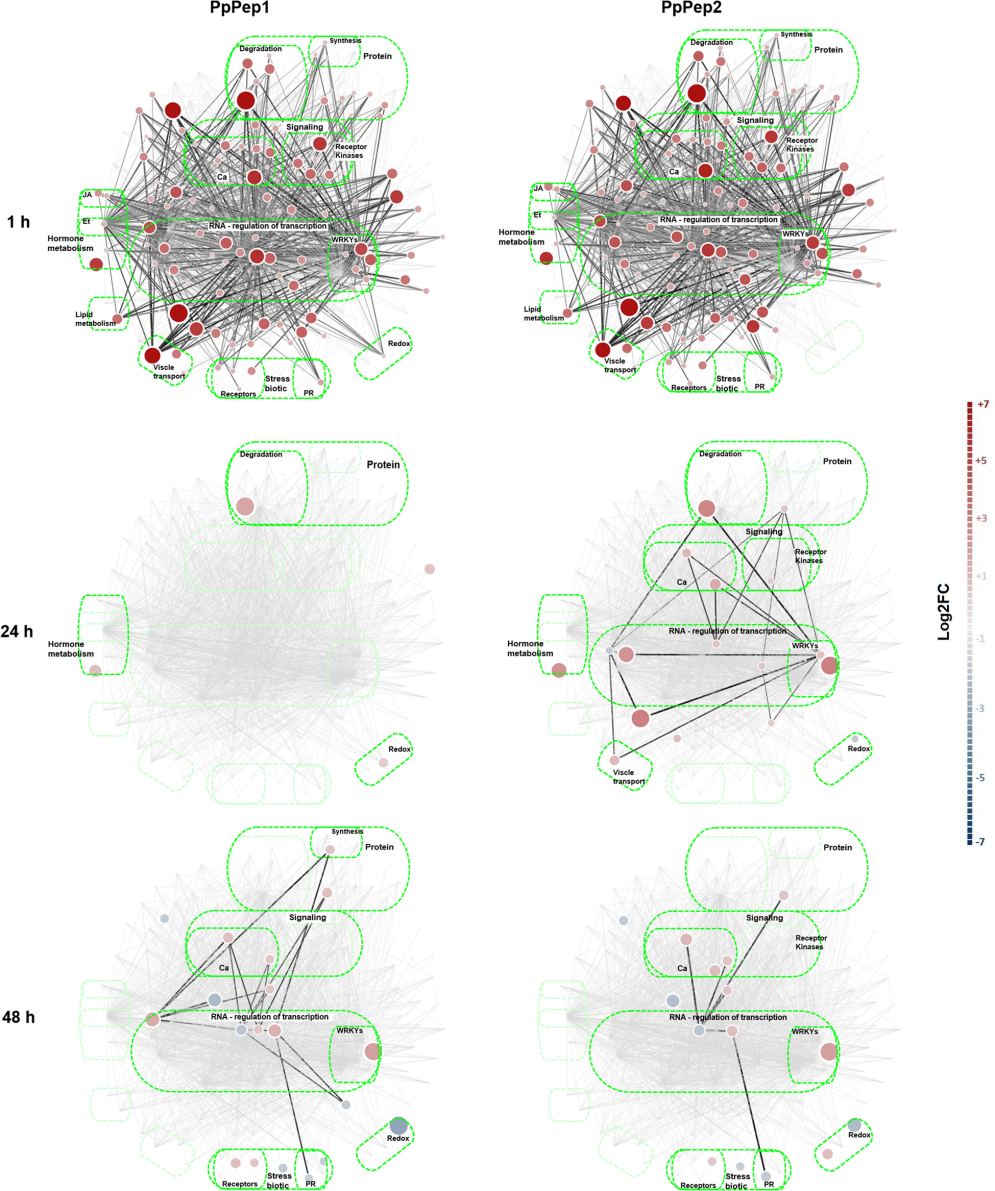
Differential network visualization of gene expression data in the context of custom *P. persica* Pep network (PEPN) using DiNAR. *P. persica* response to PpPep1 (left) and PpPep2 (right) 1, 24 and 48 h after application (top to bottom). Genes with differential expression in each condition compared to the untreated samples (adj. *p* < 0.01; |log2FC| > 1.0) are shown. Node colors correspond to the expression fold changes. Node sizes correspond to the absolute fold changes and are proportional to the custom threshold (− 7, 7). Edges represent the regulation between the components (transcription, activation, inhibition, binding or synthesis) while their color intensity depends on the differential expression of the genes connected by them (i.e. shown only if the differential expression of both genes is statistically significant). BINs containing DEG are named and framed by brighter green ovals

*P. persica* specific background knowledge network was built based upon the first neighbors of the validation-selected genes and published connections within the *Arabidopsis thaliana* comprehensive knowledge network [31]. This led to a *P. persica* Pep background knowledge network with 629 nodes and 7393 edges. To make the visualization of the transcriptomics rewiring events and the dynamics of the underlying system more exploratory and biologically informative, this initial background network was further clustered using the DiNAR sub-app. Post clustering step, the most informative cluster was selected, i.e. cluster 2. Hence cluster 2 from the *P. persica* Pep background knowledge network lastly contained 195 nodes and 1698 edges that represented the most expressive and dynamic transcripts. Relative gene expression data sets 1, 24 and 48 h after treatment with PpPep1 and PpPep2 (each compared to untreated samples) were superimposed on *P. persica* Pep background knowledge network using DiNAR. Figure 5 shows a static visualization of our peach tree dataset superimposed on cluster 2 from this network. Dynamic data, together with further information on every node (gene code) and edge connection type (e.g. transcriptional regulation) is presented in the Additional files 10 and 11.

### Comparison of the transcriptomic responses to PpPep1 and PpPep2

We used the GSEA tool for more detailed information on the differences between PpPep1 and PpPep2 inspected using DiNAR (Table 2). Note that these differences do not exceed the 6.31% (Fig. 2 and Additional file 3) of the variability in the transcriptomic data. Direct comparison of the transcriptomes of *P. persica* leaves treated with either PpPep1 or PpPep2 (Additional file 8) shows some statistically enriched BINs, mainly 1 h and 1 day after treatment. These BINs were mostly enriched at just one time point, suggesting the variations between the two peptides were temporary. More than 80% of the genes contributing to the enrichment of these BINs in the PpPep1 vs. PpPep2 comparison were overrepresented in the time course comparisons Peps response (Table 1). This suggests that differences between PpPep1 and PpPep2 result primarily from either shifted expression changes or different expression levels in the same gene set and/or gene type, with only e.g. slightly different expression of genes that have been related to environmental stresses (Table 2).

**Table 2:**
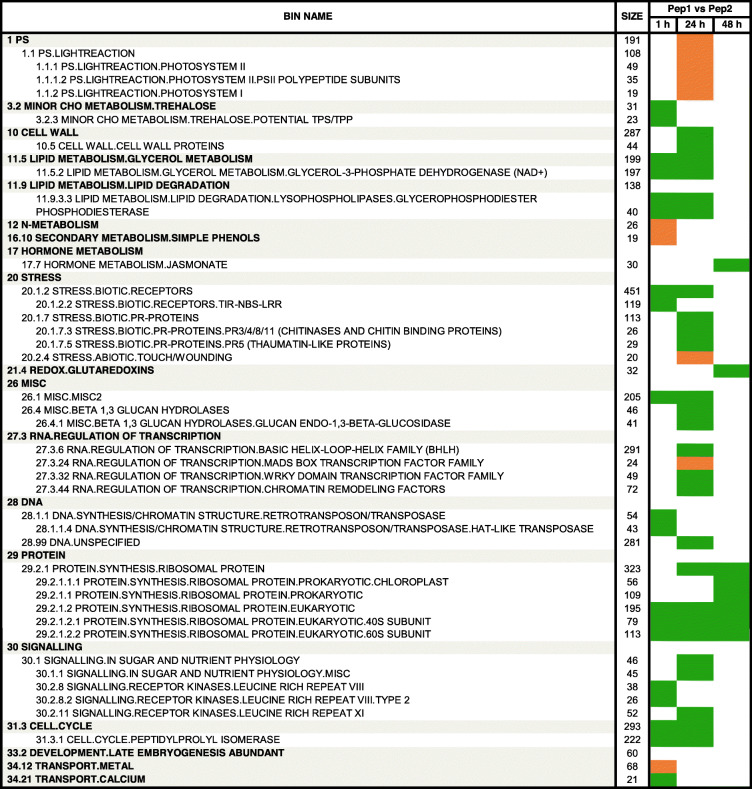
Comparison of the transcriptome response of *P. persica* leaves to PpPep1 and PpPep2

Gene set enrichment analysis was carried out using GSEA and adjusted *p* < 0.01. Orange and green indicate overexpression on PpPep1 and PpPep2 treatment, respectively, of the genes contributing to each bin. Blank cells indicate the absence of statistically significant enrichment. Size indicates the number of genes in the gene set after filtering out those not in the expression dataset. Additional file 8 shows the data for all BINs and comparisons

### Comparison of the responses to Peps and pathogens

**Fig. 6:**
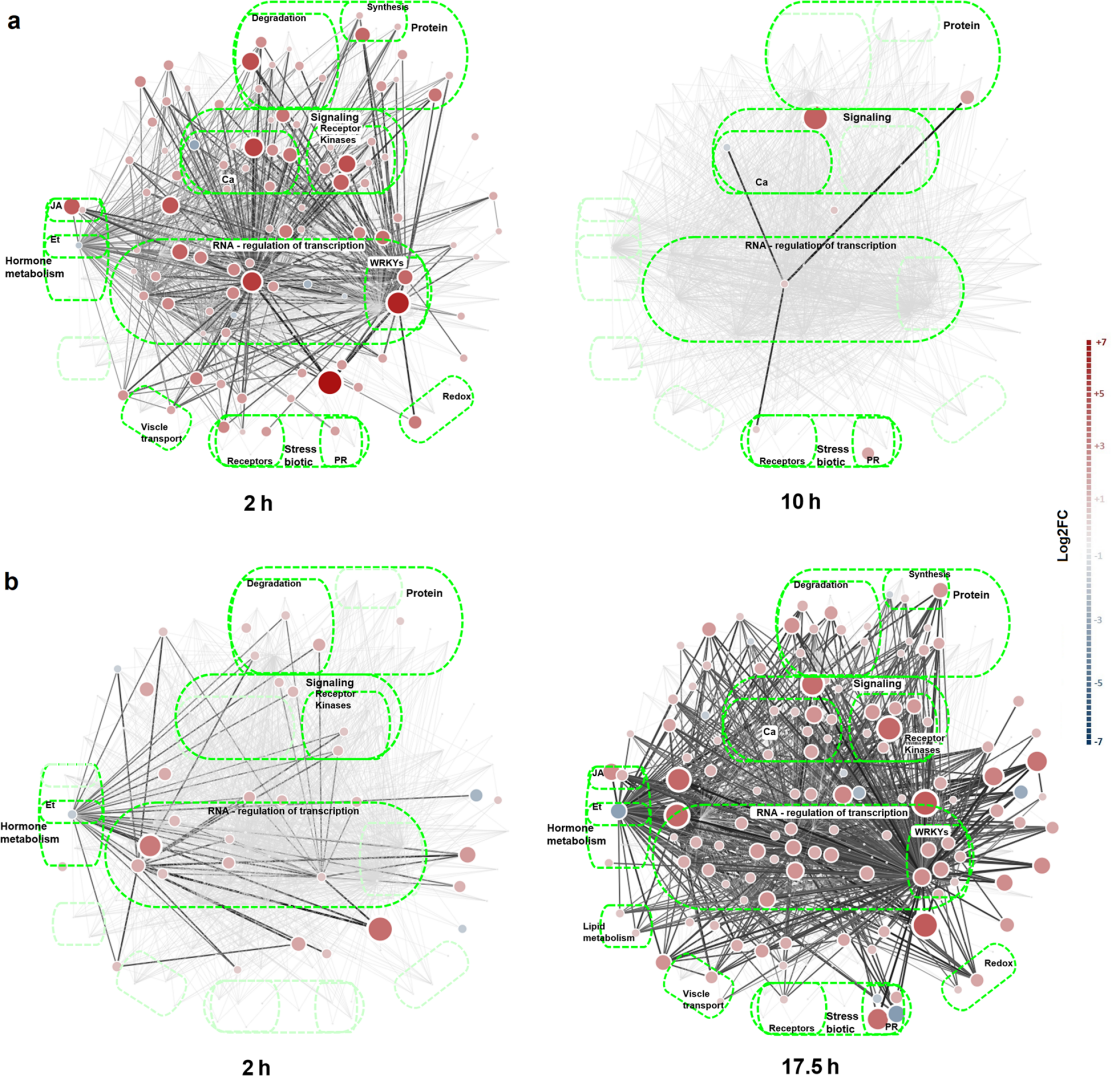
Differential network visualization of gene expression data in the context of custom *P. persica* Pep network (PEPN), cluster 2, using DiNAR. **a** Response of *A. thaliana* to AtPep2, two and 10 h after application using data published in [22] (top); **b** infection with *Pseudomonas syringae* pv. *tomato* DC3000, two and 17.5 h after pathogen inoculation using data reported in [52] (bottom). (a and b) Genes exhibiting differential expression in each condition compared to untreated samples (*p* < 0.05 and |log2FC| > 1.0) are shown. Node colors correspond to the expression fold changes. Node sizes correspond to the absolute fold changes and are proportional to the custom threshold (− 7, 7). Edges represent regulation between the components (transcription, activation, inhibition, binding or synthesis), while their color intensity depends on the log2FC. BINs containing DEG are named and shown in brighter green

Using the same background knowledge network, we compared the response dynamics to Peps and pathogen infection in DiNAR. We superimposed onto the *P. persica* Pep background knowledge network two published transcriptome datasets: (i) *A. thaliana* leaves 2 and 10 h after treatment with the compatible AtPep1 [22] and (ii) *A. thaliana* leaves 2 and 17.5 h after infection with the pathogenic bacterium *Pseudomonas syringae* pv. *tomato* DC3000 (Pst) [32]. Figure 6a, b and Additional files 12 and 13 show a visualization of the two transcriptome pattern dynamics, identification of strong differential interactions and recall of common effects. Node location and BIN representation are unique in a given context, allowing for a straightforward comparison between samples.

## Discussion

Several studies have demonstrated that pre-treatment of Peps significantly improves plants resistance to pathogens [1, 3, 6, 7, 9, 12] and we have previously determined that low doses of topical application of Rosaceae Peps efficiently enhance resistance of *Prunus* spp. to Xap infection [9] in an ex vivo system. Here we showed that PpPep1 and PpPep2 are also effective in vivo, which strengthens their candidacy for an actual environmental-friendly strategy to enhance plant resistance. The protective effect of topical application occurred at nanomolar concentrations of either PpPep1 or PpPep2, decreasing the deleterious consequences of massive Xap inoculation onto peach plants that were challenged with pathogen concentrations of 10^8^ cfu/mL, i.e. higher than expected in nature. In these extreme conditions, the infection symptoms were reduced by up to 40%. Interestingly, some indicators showed a prolongation of this protection when more moderated infections occurred. We observed a dose-dependent effect on plant protection. For PpPep2, 1 and 10 μM were the most effective doses reducing Xap symptoms 9 days after massive infection (Fig. 1). Unexpectedly, the maximum protection with PpPep1 was achieved at 0.1 μM doses, suggesting that peach has a higher sensitivity to PpPep1 perception which could be interesting for field application strategies.

We analyzed the peach response to topical application of peach Peps through RNA-Seq, which allowed us to characterize the immune triggering and provided as well detailed transcriptomics information that would facilitate identification of any unwanted effect that could rise food safety concerns.

Overall, up to 8% of the *P. persica* genes were regulated in response to PpPeps. The numbers of DEG shared under both 1 h and 24 h conditions indicated a transient transcription induction shortly after peptide application that affected up to 4% of *P. persica* genes (Fig. 3 and Additional files 5 and 6). Up to 6% of the peach genes were commonly altered after topical application of PpPep1 and PpPep2 (Fig. 3 and Additional files 5 and 6), which demonstrated a highly similar transcriptomic response up to 2 days after exposure to the peptides, especially shortly after treatment (1 h). At later stages we observed some asynchrony and variation in the specific regulated genes, still within the same functional categories (Table 2). This supports the observed differences of PpPep1 and PpPep2 in reducing Xap infection severity.

DiNAR integration and custom background knowledge network allowed us a dynamic visualization of the transcriptomes of peach leaves upon treatment with PpPeps (Fig. 5 and Additional files 10 and 11). It showed the aforementioned shared, strong response to PpPep1 and PpPep2 1 hour after application, with virtually the same network rewiring events. One day after peptide application there was deregulation of most nodes, nearly reaching control levels in the case of PpPep1 treatment. Two days after treatment the regulated gene set was largely different from that observed before. There was evident decrease in the similitude between the responses to PpPep1 and PpPep2 one and 2 days after treatment, which seemed to reflect a difference in the rhythm of gene regulation, PpPep2 being slower than PpPep1. This is in agreement with our PCA (Fig. 2 and Additional file 3) showing that 93.69% variability of our RNA-Seq results depends on the time course; and that samples of peach leaves treated with either PpPep1 or PpPep2 for a given period of time are not separated by any PC.

A gene set enrichment analysis was carried out with GSEA to describe the transcriptomic response to PpPeps in detail. It was confirmed that, during the whole-time course, both PpPep1 and PpPep2 induced major regulation in functional categories related to activation of PTI (Table 1). The identity of regulated BINs is in agreement with the described responses of Arabidopsis to its specific AtPeps [21, 22] and suggests that in general terms, Pep responses in commercial species such as peach are similar to the model species.

Pep perception occurs via the corresponding PEPRs. The peach PEPR gene family encompasses PEPR1a and PEPR1b [3, 9]. They cluster in SUBBIN 30.2.11, comprised of leucine-rich repeat receptor kinases (LRR-RK) of the subfamily XI, involved in plant development and differentiation [12]. Both results, our RNA-Seq and RT-qPCR, showed no detectable expression of PpPEPR1a or PpPEPR1b in untreated leaf blades. This is similar to the Arabidopsis AtPEPR gene family, for which a basal expression in leaves was restricted to the veins [2, 25, 33]. One hour after PpPep1 or PpPep2 treatment, strong induction of PpPEPR1a (Prupe.3G167800), but not PpPEPR1b (Prupe.3G167900), was observed. AtPEPR1 and AtPEPR2 expression overlap to some degree, but in general they have different expression patterns. AtPEPR1 has been shown to be induced by application of any AtPep, whereas AtPEPR2 transcript levels rise upon treatment with only few AtPeps [12]. Safaeizadeh and Boller [25] showed that the AtPEPR1 (but not AtPEPR2) promoter drove GUS accumulation 1 h after AtPep1 or flg22 treatment in leaves. Our results suggest that PpPEPR1a has a role in driving PpPep1 and PpPep2 signal transduction in peach tree leaves, and perhaps both PpPeps might be recognized by PpPEPR1a in leaves. Similarly, in Arabidopsis, AtPEPR1 can detect all eight AtPeps (AtPEPR2 detects only AtPep1 and AtPep2, [2]).

SUBBIN 20.1.2. (biotic stress sensing receptors) exhibited a similar pattern. Of special interest within this SUBBIN is FLS2, which recognizes a conserved N-terminal 22-amino acid sequence (flg22) of bacterial flagellin as a PAMP. PEPR and FLS2 have strong similarities in structure and target recognition, and initiate pattern-triggered immunity (PTI) in a parallel way. In addition, not only AtPeps but also flg22 have been shown to induce the expression of AtPEPRs [25]. Here we demonstrate that, similar to Arabidopsis and AtPep1, peach leaves have a rapid, transient reaction to PpPeps inducing both PpPEPR1a and PpFLS2 (Prupe.3G304400) in a similar manner.

Several receptor-like cytoplasmic kinases (RLCKs) of the subfamily VII play key roles in PTI signaling. Botrytis-induced kinase1 (BIK1) and PBS1-like1 (PBL1) directly interact with RK such as PEPR1 and FLS2 [15, 34] and contribute to resistance to pathogens and herbivores [35–37]. Other RLCK VII members have been suggested to contribute to the PTI. In peach an induction of RCLKs VII (including e.g. PBL1) expression which constitute the enriched BIN 29.4.1.57, was observed. This points towards their involvement in PpPep signal transduction similar to that described in the model species [5, 6, 26, 38].

The observed extensive transcriptional reprogramming following treatment with PpPeps included induction of genes with regulatory function, notably (i) those related to the hormone signaling, with special significance of ethylene (ET) and abscisic acid (ABA) hormone signal transduction (BINs 17.5.2 and 17.1.2, respectively); and (ii) those related to the regulation of transcription, particularly chromatin remodeling factors (BIN 27.3.44) and the WRKY transcription factor family (BIN 27.3.32). We identified 19 different WRKY transcriptional regulators with quick and transient upregulation upon persistent Pep application (Additional file 9). Proteins of this large family are characterized by the WRKY-domain and are involved in the regulation of plant resistance to a variety of pathogens in a complex regulatory network [39]. In agreement with our results, Pep sensing has been shown to involve several WRKYs e.g. WRKY33, which has been associated with AtPROPEP2 and AtPROPEP3 promoter activities [40].

In Arabidopsis and maize, PTI signaling cascade leads to the ET production in a matter of hours, as well as in a slight increase in the jasmonic acid (JA) [6, 13, 41]. In addition, Ross and colleagues [22] have described induction of ET and JA regulated genes in response to the AtPep2. Enrichment of BINs 17.5 and 17.5.2, 1 hour after the PpPep treatment of leaves, indicates that ET also plays an important role in the Pep signaling pathway in peach. These BINs contain genes responsible for ET biosynthesis e.g. 1-aminocyclopropane-1-carboxylate synthase and oxidase [42, 43]; and numerous ET-responsive transcription factors (ERF), which are the last layer of regulation of JA/ET-responsive defense genes [44] and can play a role in the resistance to necrotrophic pathogens. In contrast to MAMPs, AtPeps do not have major effects on SA synthesis but they co-activate both SA and JA/ET pathways in Arabidopsis*,* as they also trigger expression of several SA-induced genes [21, 22]. Our peach results and the PpPep system showed no enrichment of functional categories specifically linked to SA metabolism and signaling pathways. However, NDR1, a gene that belongs to the SA metabolism BIN and is required for antibacterial immunity [45], was significantly upregulated 1 h after treatment with any PpPep. In addition, the enriched BIN 20.1.7.1 gathered genes such as PR1 and PR4 that are considered markers for SA and ET/JA signaling, respectively, overexpressed 2 days after Pep exposure. The nonexpressor of PR genes1 (NPR1) (enriched BIN 20.2.1), which mediates resistance to biotrophic and hemi-biotrophic pathogens, is the main receptor of SA [46]. Abscisic acid (ABA) has multiple roles in abiotic and biotic stresses. It is primarily considered a negative regulator of disease resistance, through interference with signaling pathways of several other stress-response hormones including JA and ET [47, 48]. In peach leaves, regulation of BIN 17.1.2 containing genes involved in ABA signal transduction suggests participation of ABA on fine tuning the response to PpPeps.

Here we found quick and transient upregulation of calcium signaling and calcium transport proteins (BINs 30.3 and 34.21, respectively). Calcium signaling is part of PTI, acting immediately downstream from FLS2 and BAK1. Changes in cytosolic Ca^2+^ concentration ([Ca^2+^]cyt), together with ROS and electrical signals form signaling networks that drive local and systemic defense responses [49]. Toyota and colleagues [50] reported that glutamate (Glu) can act as a DAMP that is sensed by Glu receptor like (GLR) ion channels to trigger an increase in [Ca^2+^]cyt, propagating the defense response to distant organs. In peach leaves, overexpression of GLR (BIN 30.1) and other genes related to [Ca^2+^]cyt (e.g. various calcium-dependent protein kinases, calmodulin proteins, BIN 30.3 and 17.5) upon Pep treatment suggests that Peps might act as DAMPs and modulate both local and systemic signaling through Ca^2+^ and perhaps Glu [51].

Plant disease resistance (R) proteins recognize effectors specifically secreted by adapted pathogens to suppress PTI. We found upregulation of several BINs encompassing R proteins: BIN 30.2 (RK with signaling function); BIN 20.1.2 (the two SUBBINs of biotic stress sensing receptors) and BINs 11.9 and 27.3.44 (lipid degradation and chromatin remodeling factors such as rust resistance kinases). Most R proteins have an LRR motif and many also have a nucleotide binding site (LRR-NBS). Examples of LRR-RLK R proteins that are part of the peach response to Peps are ZAR1, Xa21 and RCH1. ZAR1 and Xa21 recognize bacterial effectors from *Pseudomonas syringae* [52] and *X. oryzae* pv. *oryzae* (Ax21, [53]) whereas RCH1 recognizes a fungal effector from *Colletotrichum higginsianum* [54]. Among the LRR-NBS R proteins induced by PpPeps in peach, four are leaf rust 10 disease-resistance locus receptor-like protein kinases (LRK10L). In wheat, LRK10L proteins recognize effector proteins of the basidiomycete *Puccinia striiformis* f. sp. *tritici* Eriks & Henn, which causes stripe rust, and drives ETI [55]. Noteworthy is that genes belonging to the same BINs were overexpressed in *P. persica* 30 min to 3 h after infection with pathogens such as Xap [56]. Persistent exposure to Peps triggers the synthesis of a battery of R proteins, building up the capacity to establish ETI in response to a variety of virulent pathogens that include bacteria and fungi.

PTI also involves reactions occurring within days, e.g. mainly cell wall remodeling and fortification; and fast synthesis of pathogenesis related proteins (PR) [23, 24] as well. We found that Peps triggered changes in the cell wall related genes (BIN 10) and the biotic stress PR proteins (BIN 20.1.7.1 and 20.7.1.5) 24 and 48 h after application.

The dynamics of the transcriptomic response of *P. persica* leaves to PpPep1 and PpPep2 were compared to other immune response datasets through DiNAR (Additional files 12 and 13). Similar to *P. persica*, there is a strong transcriptional response in Arabidopsis shortly after applying a compatible Brassicaceae Pep. This mainly involves upregulation of genes in the same BINs, which in turn are related to the described PTI. Even if data correspond, respectively, to 1 h and 2 h PpPep1, PpPep2 and AtPep2 all activate a number of nodes which, in consequence, could be considered candidate indicators of plant responses to Peps. Some examples are the stress related transcription factors ZAT10, ZAT12, WRKY46, LRK10L1.2, the calcium binding proteins CML40 and CML46, the ethylene responsive factors EIN3 and ERF011; the RLKs PBL19 (membrane) and RLK RPP13-like protein 4 (cytoplasmic); and the immune response regulators HSPRO2 (nematode resistance protein), subtilisin-like protease SBT3.3 and F-box protein At1g61340. Ten hours after AtPep2 treatment Arabidopsis virtually had the basal transcriptome, which parallels the peach response to peptides around 1 day after application.

This was in contrast to the dynamics caused by pathogen attack. Pst had a similarly intensive effect on the transcriptome of the plant only after 17.5 h. There were 35 and 127 regulated nodes, two and 17.5 h, respectively, after Pst infection. This might be due to progress of the infection, which makes the response of different cells asynchronous and the delay in multiplication of the pathogen that then triggers the response. However, Pst attack and Pep treatment largely affected the same BINs. 64% nodes regulated by Pst also reacted to Pep, and 84% nodes regulated by Pep were altered by pathogen attack. This shows that, even if the precise conditions of the compared experiments are different there is an evident parallelism in the transcriptome responses to Pst and AtPep1.

## Conclusion

Here we showed the protective effect of topical application at nanomolar concentrations of either PpPep1 or PpPep2, decreasing the deleterious consequences of massive *Xanthomonas arboricola* pv. *pruni* inoculation onto peach plants that were challenged with pathogen concentrations of 10^8^ cfu/mL, i.e. higher than expected in nature. In these extreme conditions, the infection symptoms were reduced by up to 40%.

On characterization of the peach response to topical application of either PpPep1 or PpPep2, in the concentrations giving major protection against Xap, we observed clear PTI activation. This parallels the reported response of Arabidopsis to AtPep1 and confirms that compatible Peps enhance peach basal immunity, so decreasing pathogen effects. The PpPep common transcriptomic response represents 94% observed variability and this includes PEPR1a, suggesting a role for both PpPep1 and PpPep2 mediated activation of PTI in peach leaves. The similarity of PpPep responses is higher shortly after treatment (1 h), with some asynchrony at later stages and some variation in the specific regulated genes, always within the same functional categories. The effect of PpPep1 in reducing Xap infection severity peaked at 10-fold lower concentrations than for PpPep2, suggesting that peach leaves have a higher sensitivity to PpPep1 perception and response. The 6% transcriptome variability in PpPep1 and PpPep2 may be associated with different peptide optimal doses, affinity to the receptor and coreceptors, and persistence in plant leaves.

DiNAR proved to be an intuitive, visual and easy platform to analyze the main results of transcriptomics assays and to compare samples representing different conditions (e.g. species, treatments). The initial selection of the most variable transcripts, custom background network construction and subsequent clustering, proved to be sufficient approach to identify general similarities and major differences between the samples. As an alternative, GSEA uses the full information and is most adequate for detailed comparisons and identification of minor differences in samples.

In this prospective assay, PpPeps was shown to protect peaches from extremely high Xap doses while inducing transcriptomic reprogramming similar to PTI, the natural response to pathogen attack. We found no evidence suggesting that PpPep topical application could rise any food safety concern, so PpPeps seem to be plausible candidate molecules for use in natural and environmental-friendly agronomic plant protection strategies.

## Materials and methods

### Plant and bacterial material

*Prunus persica* var. Big Top (peach) juvenile plants were produced using in vitro technology and grown in individual small pots by a professional grower (Agromillora Iberia S.A., Barcelona, Spain). Prior to experiments they were acclimatized for 2 weeks in a glasshouse (21 °C, 16/8 h light/dark photoperiod). GF-677, a cross of *P. persica* and *P. dulcis* was used in some experiments, as described in [9].

*Xanthomonas arboricola* pv. *pruni* (Xap) strain CFBP 5563 (*Collection Française de Bactéries Phytopathogènes*, Angers, France) [57] was used to infect peach as described in [9]. A dose of 10^8^ cfu/mL, suspended in sterile water, was prepared immediately before use and the concentration was verified by plate counting. Experimental research was carried out in compliance with relevant national, and international guidelines and legislation, notably Regulation (EU) 2016/2031 of the European Parliament of the Council of 26 October 2016.

### In vivo peptide assays

PpPep1 (EVAASSRVVRQPITTGGGGQIN, full-length mature peptide of MW600836) and PpPep2 (YVQRITLRAARPEISTGSGAQTN, full-length mature peptide of MW600837) [9] were chemically synthesized (Caslo ApS, Lyngby, Denmark) with purity above 95% and the identity confirmed by MALDI-TOF. The stock solution was prepared at 1 mM in double-distilled water, and end concentrations of 10, 1, 0.1 and 0 (mock control) μM were prepared prior to use. For treatment with Peps, the five youngest fully expanded leaves of each plant were selected and labelled; and the corresponding Pep was sprayed onto both abaxial and adaxial leaf surfaces. Plants were incubated under standard conditions in the glasshouse. There were three biological replicates of nine plants per treatment.

For RNA-Seq and RT-qPCR analysis, treated leaves were detached 1, 24 and 48 h after peptide treatment, the central vein was cut out and the leaf blades were immediately frozen in liquid nitrogen for subsequent RNA extraction.

For peptide elicitor activity assays, 650 μL of a freshly prepared 10^8^ cfu/mL Xap suspension was sprayed onto five adult leaves of each plant 24 h after treatment, with either PpPep1 or PpPep2. The progress of bacterial spot infection was monitored weekly over for three to 4 weeks. For the disease severity index, a 0-to-6 interval scale was used, corresponding to the level of leaf area affected: 0, 1–3, 4–8, 9–15, 16–25, 26–45 and > 45% (leaf abscission), respectively (Additional file 14, [58]). Disease severity (*S*) was calculated for each plant according to the equation: S = [(${\sum}_{n=1}^N$ I_n_)/N × 6] × 100, where *I*_*n*_ is the severity index for each leaf, *N* is the number of leaves per plant, and *6* is the maximum severity index value in the scale. Kruskal-Wallis post-hoc pairwise comparisons (IMB SPSS Statistics 25, *p* < 0.05) were used to analyze the severity. The assays were repeated twice with different batches of plants.

### RNA extraction and Illumina sequencing

RNA was extracted from a 200 mg aliquot of ground leaf sample using a two-step TRIzol-based procedure (Invitrogen Life Technologies, Carlsbad, CA, USA) followed by DNAse I (Ambion, Grand Island, NY, USA) digestion of remaining DNA. For Illumina sequencing, RNA was further purified using RNeasy MinElute Cleanup Kit (Qiagen, Sollentuna, Sweden) according to the manufacturer’s instructions. RNA concentration was estimated through absorbance at 260 nm using a NanoDrop ND1000 spectrophotometer (Nanodrop Technologies, Wilmington, DE, USA).

RNA-Seq was carried out at the National Centre for Genomics Analysis (Barcelona, Spain). Total RNA from *Prunus persica* was quantified by Qubit® RNA BR Assay kit (Thermo Fisher Scientific) and the RNA integrity was estimated by using RNA 6000 Nano Bioanalyzer 2100 Assay (Agilent).

The RNA-Seq libraries were prepared with KAPA Stranded mRNA-Seq Illumina® Platforms Kit (Roche) following the manufacturer’s recommendations. Briefly, 500 ng of total RNA was used for the poly-A fraction enrichment with oligo-dT magnetic beads, following the mRNA fragmentation. The strand specificity was achieved during the second strand synthesis performed in the presence of dUTP instead of dTTP. The blunt-ended double stranded cDNA was 3’adenylated and Illumina platform compatible adaptors with unique dual indexes and unique molecular identifiers (Integrated DNA Technologies) were ligated. The ligation product was enriched with 15 PCR cycles and the final library was validated on an Agilent 2100 Bioanalyzer with the DNA 7500 assay.

The libraries were sequenced on HiSeq 2500 (Illumina) with a read length of 2x76bp + 8 bp + 8 bp using TruSeq SBS Kit v4 (Illumina). Image analysis, base calling and quality scoring of the run were processed using the manufacturer’s software Real Time Analysis (RTA 1.18.66.3).

### Bioinformatics analysis

RNA-Seq reads were mapped to the *Prunus persica* NCBIv2 reference genome using the STAR v2.5.3a software with ENCODE parameters from long RNA [59]. Gene quantification was performed with RSEM version 1.3.0 with default parameters using the *Prunus persica* NCBIv2.38 annotation version. Differential expression analysis was performed with R package DESeq2 version 1.20.0 [60] with default parameters. Genes were considered significant with FDR < 0.01 and |log2FC| > 1.

The annotation of *P. persica* genes based on their corresponding orthologues in *Arabidopsis thaliana* was performed using the Genome Database for Rosaceae [61]. Overlapping DEG in response to PpPep1 and PpPep2 were represented using the BioVenn application [62]. Agglomerative Hierarchical Clustering (Euclidean distance, ward. D agglomeration method) of 2542 selected genes (log2FC along 6 comparisons) was conducted in R [63] using *gplots* library [64].

Gene set enrichment analysis was carried out with GSEA v4.0.1 [65]. RNA-Seq ranked gene list was input to GSEA [30] and the settings applied were: ‘gene set permutation’ as statistical significance of the enrichment score, signal-to-noise ratio as the ranking metric, settling 1000 permutations per test and excluding sets not within 15–500. The resulting pathways were selected using a FDR *Q* value threshold < 0.01 and ranked using Normalized Enrichment Score (NES).

Transcriptomic time-point dynamic changes were visualized using DiNAR [66]. A custom background knowledge network was constructed using orthologue gene information (PLAZA v4.0, [67]) based translation of *A. thaliana* network [31] to *Prunus persica* network and further clustered using the multi-level modularity optimization algorithm using DiNARs’ subapps. Peach gene IDs within one orthologue group were prioritized based on their expression using the accompanying DiNARs script for ID prioritization. To facilitate interpretation of the DiNAR output, we manually organized the coordinates of the background network nodes (Cytoscape v3.7.2, [68] on the basis of their functional category prescribed BINs as defined by the MapMan resource for gene functional annotations [69, 70]. Nodes associated to the same BIN were aligned in the neighborhoods, and the most relevant BINs encompassing the nodes with the highest regulation were displayed in the *P. persica* Pep background network. Additional files 15 and 16 compiles the input data of this network (also available on https://github.com/NIB-SI/DiNAR/tree/master/PEPN).

### RT-qPCR analysis

Reverse transcription and real-time PCR (RT-qPCR) was performed to validate the RNA-Seq results and to conduct the additional gene expression analysis. For specific qPCR optimization, the selected genes were PCR-amplified from cDNA, synthesized from untreated leaf samples, and PCR products were cloned using the pSpark DNA cloning system (Canvax, Córdoba, Spain). The reaction conditions were as follows: 2 min at 94 °C; 10 cycles of 15 s at 94 °C, 30 s at the appropriate annealing temperature (Additional file 17) and 45 s at 72 °C; 20 cycles of 15 s at 94 °C, 30 s at the same annealing temperature and 45 s, plus an additional 5 s for each successive cycle at 72 °C; and a final extension of 7 min at 72 °C.

cDNA was synthesized from RNA samples using High Capacity cDNA Reverse Transcription Kits (Thermo Fisher Scientific Inc.). The qPCRs were performed in a final volume of 20 μL, containing 1X SYBR Green PCR Master Mix (Thermo Fisher Scientific Inc.), the appropriate concentrations of primers (Merck KGaA, Darmstadt, Germany) (Additional file 17) and 1 μL cDNA. The reaction conditions were as follows: 10 min at 95 °C for initial denaturation; 50 cycles of 15 s at 95 C and 1 min at 60 °C; and a final melting curve program of 60–95 °C with a heating rate of 0.5 °C/s. Melting curve analyzes produced single peaks, with no primer-dimer peaks or artefacts, indicating the reactions were specific. Average expression stability (M-value) of two described reference genes for *P. persica* -*TEF2* and *UBQ*- [9, 71] were determined by the GeNorm v3.4 algorithm [72]. Both M-values were < 0.5 and *TEF2* was used for normalization in rear assays. The comparative Ct (ΔΔCt) method and T-test or the corresponding non-parametric analysis were executed with Genex v.4.3.1 software for differential expression analysis, using adjusted *p* value of 1% as threshold.

## Supplementary Information

**Additional file 1.** Quality control and mapping statistics of *Prunus persica* RNA-Seq analyses using Illumina.

**Additional file 2.** RNA-Seq results were validated by RT-qPCR. Identification and GO classification of the 19 selected genes and log2FC values obtained for the same samples through both techniques. Cell colors distinguish statistically significant regulated genes (blue, down-regulated; red, up-regulated) from non-significant, shown in white (adj. *p* < 0.01, |log2FC| > 1.0 for RNA-Seq; adj. *p* < 0.05 for RT-qPCR). Graph on the right shows simple linear regression between log2FC values using the two techniques with correlation coefficient of 0.92.

**Additional file 3.** Interactive principal component analysis (PCA) of regularized–logarithm normalized expression data from the 500 genes showing the highest variance (DESeq2 1.20.0 package [60]) conducted in R [63] using scatter plot 3d library [73]. Three principal components, PC1, PC2 and PC3, with Eigenvalues above 1 explained 83.84%, 7.04% and 2.81% of the overall variability, respectively. The peptide treatments are shown in different colors: tan, time zero (t0); turquoise, 1 h PpPep1 (P1t1); light green, 1 h PpPep2 (P2t1); blue, 24 h PpPep1 (P1t24); pink, 24 h PpPep2 (P2t24); orange, 48 h PpPep1 (P1t48); yellow, 48 h PpPep2 (P2t48). Three biological replicates per sample are shown.

**Additional file 4.** RT-qPCR analysis of the same 19 genes used to validate RNA-Seq results (where the expression is regulated by application of PpPeps, Additional file 2), in mock samples taken at the 0, 1, 24 and 48 h time-points (*p* > 0.01, IBM SPSS statistics 25).

**Additional file 5.** Table of number and percentages of differentially expressed genes (DEG) in response to PpPep1 and PpPep2 at 1, 24 and 48 h after peptide application, with adjusted *p*-value cut-off 0.01. Contrasts are defined in the first column, where t stands for time, P for Pep, ‘_’ separates the conditions and ∩ for intersecting data between comparisons. DEG percentages are calculated according to the total number of genes in the *P. persica* genome.

**Additional file 6.** Venn diagrams representing overlapping differentially expressed genes in response to PpPep1 (left panel) and PpPep2 (middle panel), as well as genes commonly regulated in both treatments (right panel). 1h: 1 h vs. control; 24 h: 24 h vs. 1 h; 48 h: 48 h vs. 24 h; ∩: intersection i.e. common DEGs between comparisons; red numbers: upregulated genes; blue numbers: downregulated genes.

**Additional file 7.** Interactive heat map showing relative expression levels of genes that are regulated in at least one comparison (adj. *p* < 0.01, |log2FC| >1.0): P1t1 vs. t0, P2t1 vs. t0, P1t24 vs. t1, P2t24 vs. t1, P1t48 vs. t24, and P2t48 vs. t24. P1: treatment with PpPep1; P2: treatment with PpPep2; t stands for time and is given in hours; and the dash corresponds to vs.. The rainbow color scale represents highly positive to highly negative log2FC (7 to -7), white corresponding to 0. Gene codes are displayed on the left. Top right menu helps surfing the plot, zooming interesting areas or identifying gene codes for each expression value.

**Additional file 8.** Interpretation of the transcriptomic response of *P. persica* leaves to treatment with PpPeps. Gene set enrichment analysis using GSEA and *p* < 0.01 in time and peptide comparisons (left and right, respectively). In time comparison, red indicates upregulation and blue downregulation of the genes contributing to each BIN in the first vs. the second time condition in the upper row. In peptide comparison, orange and green indicate overexpression in PpPep1 and PpPep2, respectively, of the genes contributing to each bin. Bright red, blue, orange or green indicate statistically significant enrichment. Light colors indicate the absence of statistically significant enrichment. Size indicates the number of genes in the gene set after filtering out those not in the expression dataset.

**Additional file 9.** Detailed data of gene set enrichment analysis using GSEA and adjusted *p* < 0.01 in time comparisons. For every statistically enriched bin, all genes contributing to the enrichment are listed, as well as expression, description traits and additional GSEA ranking information.

**Additional file 10.** Dynamic visualization of *Prunus persica* response to PpPep1 from RNA-Seq experimental data on DiNAR application, *P. persica* Pep network (PEPN). Only differentially expressed genes are visualized (adj. *p* < 0.01, |log2FC| >1.0). Dynamic changes in gene expression after 1, 24 and 48 hours vs. non-treated samples are shown. Node colors correspond to gene regulation (red, upregulated and blue downregulated). Node sizes correspond to absolute log2FC values and are related to the maximum value in each time condition. Time points scale is at the bottom.

**Additional file 11.** Dynamic visualization of *Prunus persic*a response to PpPep2 from RNA-Seq experimental data on DiNAR application, *P. persica* Pep network (PEPN). Only differentially expressed genes are visualized (adj. *p* < 0.01, |log2FC| >1.0). Dynamic changes in gene expression after 1, 24 and 48 hours vs. non-treated samples are shown. Node colors correspond to gene regulation (red, upregulated and blue downregulated). Node sizes correspond to absolute log2FC values and are related to the maximum value in each time condition. Time points scale is at the bottom.

**Additional file 12.** Dynamic visualization of *A. thaliana* response to AtPep2 from microarray experimental data [22] on DiNAR application, *P. persica* Pep network (PEPN). Only differentially expressed genes are visualized (adj. *p* < 0.05, |log2FC| >1.0). Dynamic changes in gene expression after 2 and 10 hours vs. on-treated samples are shown. Node colors correspond to gene regulation (red, upregulated and blue downregulated). Node sizes correspond to absolute log2FC values and are related to the maximum value in each time condition. Time points scale is at the bottom.

**Additional file 13.** Dynamic visualization of *A. thaliana* response to *Pseudomonas syringae* pv. tomato DC3000 RNA-Seq experimental data [66, 74] on DiNAR application, P. persica Pep network (PEPN). Only differentially expressed genes are visualized (adj. *p* < 0.05, |log2FC| >1.0). Dynamic changes in gene expression following 0, 2, 3, 4, 6, 7, 8, 10, 11, 12, 14, 16 and 17.5 hours vs. mock samples are shown. Node colors correspond to gene regulation (red, upregulated and blue downregulated). Node sizes correspond to absolute log2FC values and are related to the maximum value in each time condition. Time points scale is at the bottom.

**Additional file 14.** Bacterial spot disease severity was determined by assessing Xap infected leaves using a 0-to-6 interval scale according to percent leaf area affected [58]. Disease severity (S) was calculated for each plant according to the indicated formula, where *In* is the severity index for each leaf, *N* is the number of leaves per plant, and *6* is the maximum severity index value in the scale.

**Additional file 15.** Nodes and edges input information needed for visualization of *P. persica* Pep DiNAR network (PEPN) on DiNAR. These databases were built using peach to *A. thaliana* translation based on orthologue gene information.

**Additional file 16.** Edges input information needed for visualization of *P. persica* Pep DiNAR network (PEPN) on DiNAR. These databases were built using peach to *A. thaliana* translation based on orthologue gene information.

**Additional file 17.** Primers used in RT-qPCR assays and their properties, including those targeting 19 genes selected for RNA-Seq validation and two described *P. persica* reference genes (*TEF2* and *UBQ*, [9, 71]).

## Data Availability

The datasets supporting the conclusions of this article are publicly available in the Gene Expression Omnibus (GEO) repository, record GSE161802 (https://www.ncbi.nlm.nih.gov/geo/query/acc.cgi?acc=GSE161802).

## Abbreviations

Pep: Plant elicitor peptide
PROPEP: Pep precursor
PEPR: Pep receptor
LRR: Leucine rich repeat domain
PTI: Pattern-triggered immunity
DiNAR: Differential Network Analysis in R
PEPN: *P. persica* Pep background network
