# Supplementary figures and images for: Prunus persica plant endogenous peptides PpPep1 and PpPep2 cause PTI-like transcriptome reprogramming in peach and enhance resistance to Xanthomonas arboricola pv. pruni
Source: BMC Genomics. 2021 May 18;22:360. doi: 10.1186/s12864-021-07571-9 (PMC8132438; doi:10.1186/s12864-021-07571-9)

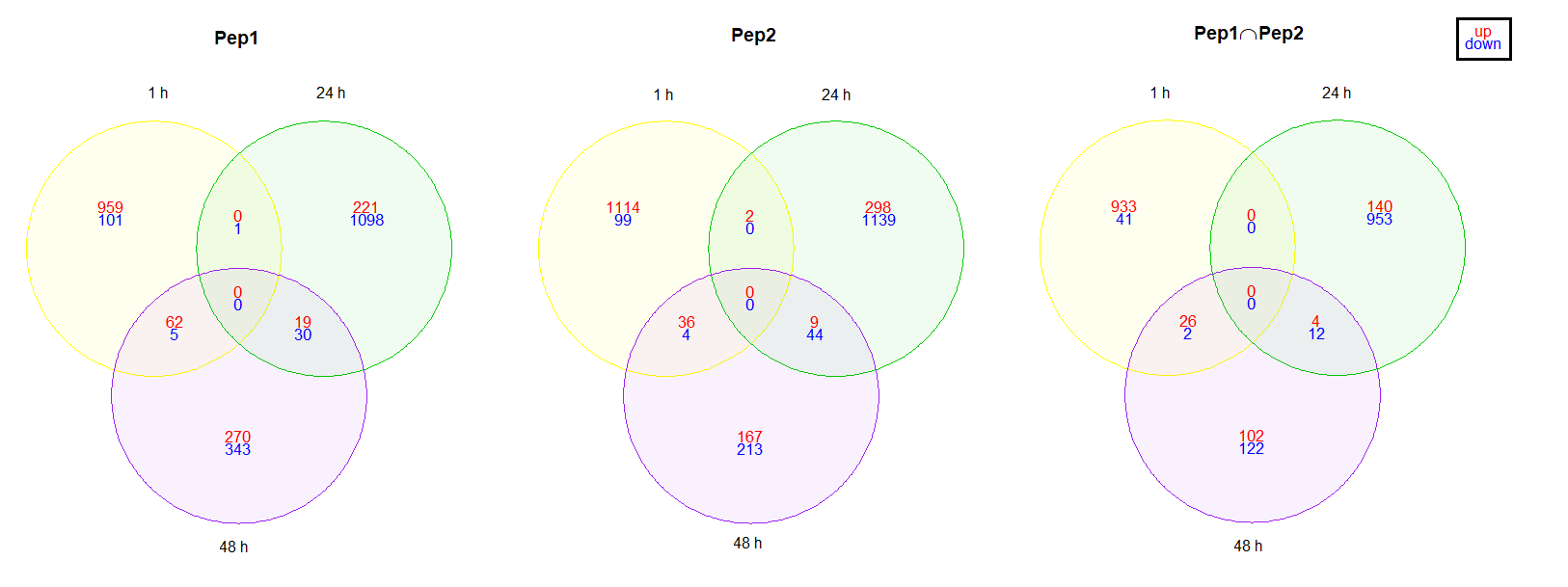

Supplement: Supplementary file 6 — Additional file 6. Venn diagrams representing overlapping differentially expressed genes in response to PpPep1 (left panel) and PpPep2 (middle panel), as well as genes commonly regulated in both treatments (right panel). 1h: 1 h vs. control; 24 h: 24 h vs. 1 h; 48 h: 48 h vs. 24 h; ∩: intersection i.e. common DEGs between comparisons; red numbers: upregulated genes; blue numbers: downregulated genes. [file 12864_2021_7571_MOESM6_ESM.tif]

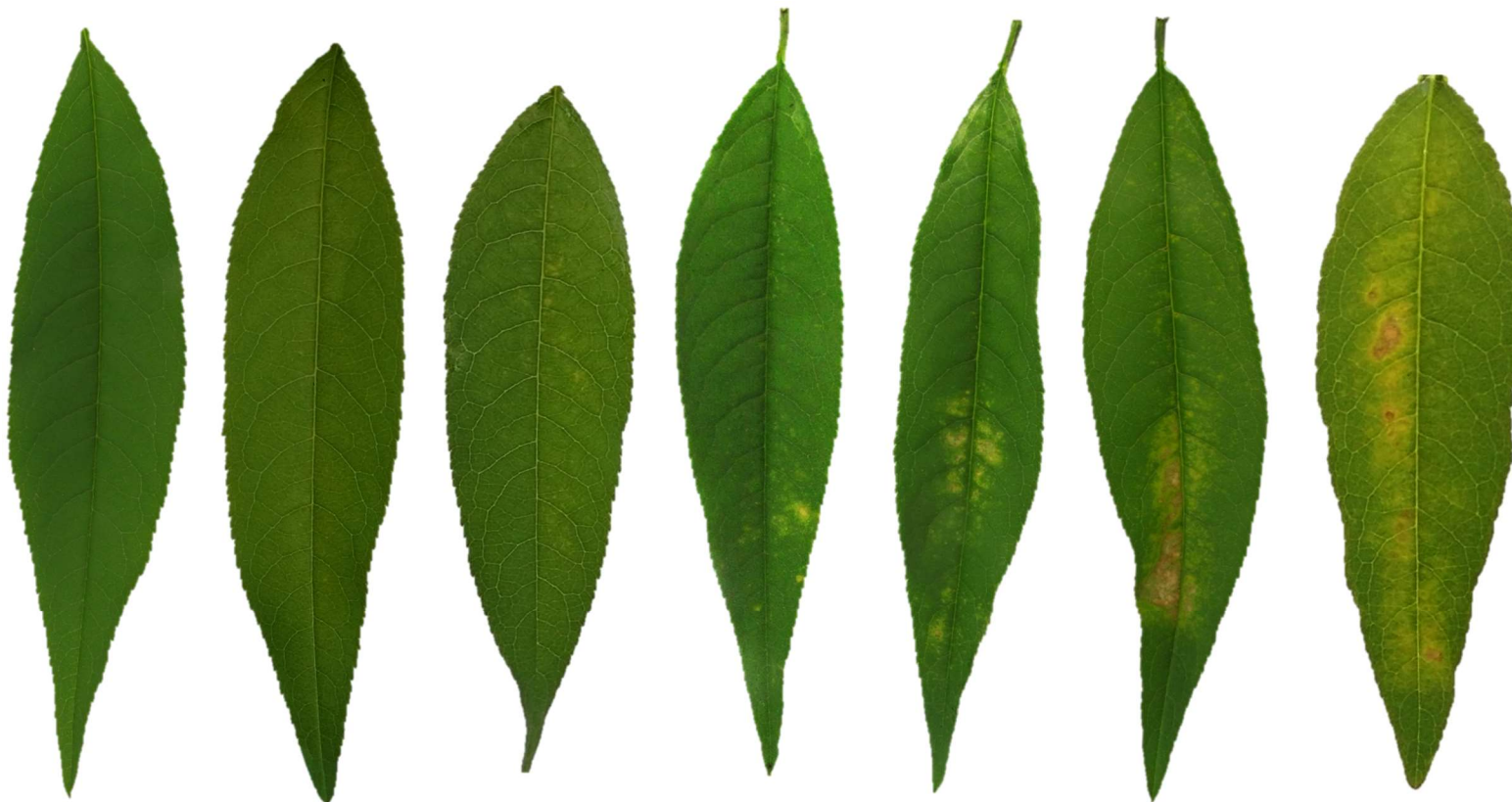

Severity index  
% lesion area

0  
0

1  
1-3

2  
4-8

3  
9-15

4  
16-25

5  
26-45

6  
> 45%

$$S = [(\sum_{n=1}^N I_n) / (N \times 6)] \times 100$$

Supplement: Supplementary file 14 — Additional file 14. Bacterial spot disease severity was determined by assessing Xap infected leaves using a 0-to-6 interval scale according to percent leaf area affected [58]. Disease severity (S) was calculated for each plant according to the indicated formula, where In is the severity index for each leaf, N is the number of leaves per plant, and 6 is the maximum severity index value in the scale. [file 12864_2021_7571_MOESM14_ESM.pdf]
