# Supplementary material for: Prunus persica plant endogenous peptides PpPep1 and PpPep2 cause PTI-like transcriptome reprogramming in peach and enhance resistance to Xanthomonas arboricola pv. pruni
Source: BMC Genomics. 2021 May 18;22:360. doi: 10.1186/s12864-021-07571-9 (PMC8132438; doi:10.1186/s12864-021-07571-9)

PEPN
Experimental data: GSE56094
Subset: *Pseudomonas syringae* pv. tomato DC3000 vs Mock

Cluster: 2 [minDegree: 1]
n: 195 e: 1709

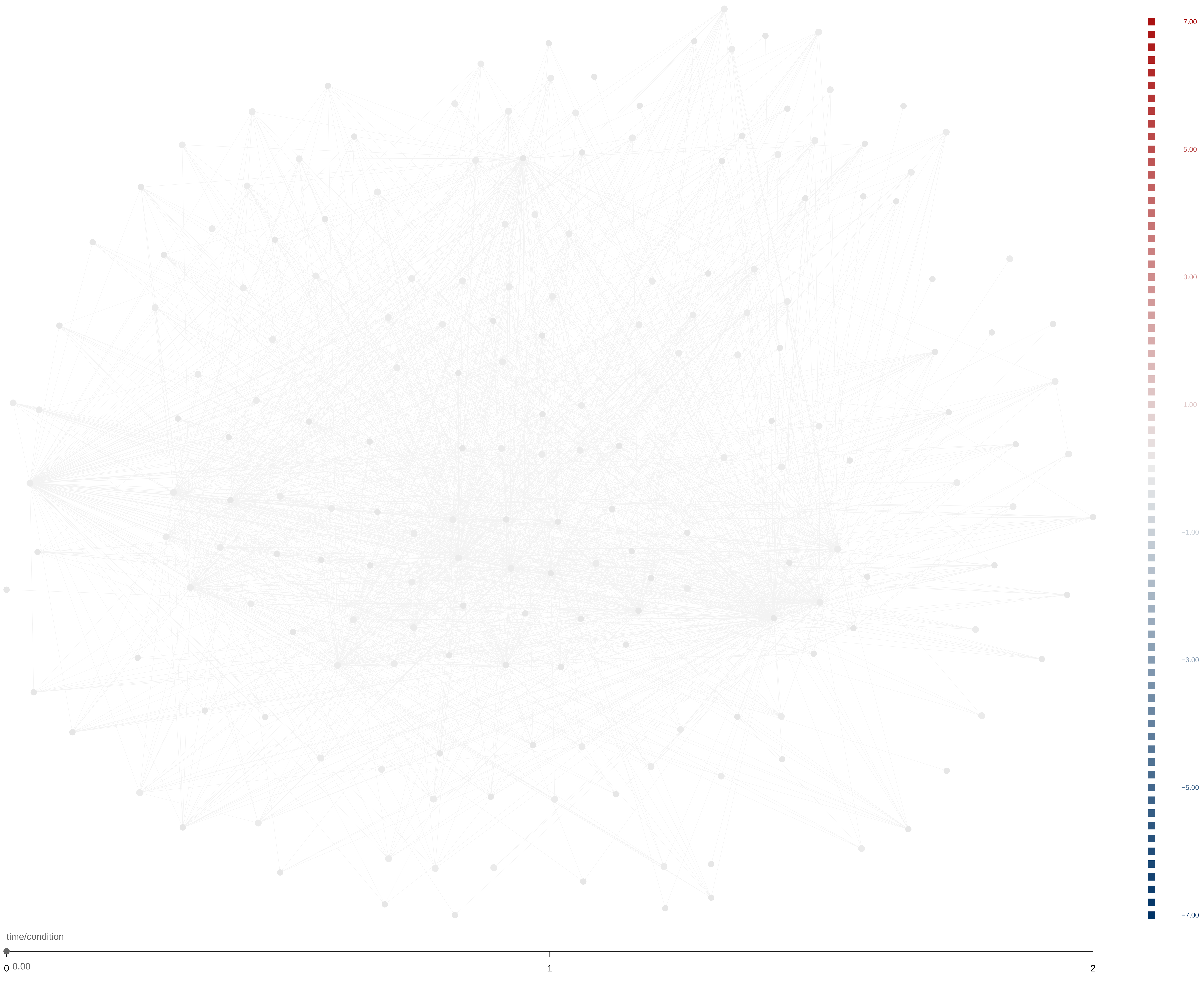

Supplement: Supplementary file 12 — Additional file 12. Dynamic visualization of A. thaliana response to AtPep2 from microarray experimental data [22] on DiNAR application, P. persica Pep network (PEPN). Only differentially expressed genes are visualized (adj. p < 0.05, |log2FC| >1.0). Dynamic changes in gene expression after 2 and 10 hours vs. on-treated samples are shown. Node colors correspond to gene regulation (red, upregulated and blue downregulated). Node sizes correspond to absolute log2FC values and are related to the maximum value in each time condition. Time points scale is at the bottom. [file 12864_2021_7571_MOESM12_ESM.pdf]
